# Blocking prostanoid receptors switches on multiple immune responses and cascades of inflammatory signaling against larval stages in snail fever

**DOI:** 10.1007/s11356-022-20108-1

**Published:** 2022-04-09

**Authors:** Sameh Saber, Suliman Y. Alomar, Galal Yahya

**Affiliations:** 1 Department of Pharmacology, Faculty of Pharmacy, Delta University for Science and Technology, Gamasa, Egypt; 2 Department of Zoology, College of Science, King Saud University, P.O. Box 2455, Riyadh, 11451 Saudi Arabia; 3 Department of Microbiology and Immunology, Faculty of Pharmacy, Zagazig University, Al Sharkia, 44519 Egypt

**Keywords:** Immune responses, Inflammatory signaling, Larval stages, PGE2, Prostanoid receptors, Snail fever

## Abstract

Schistosomiasis, also known as snail fever or bilharziasis, is a worm infection caused by trematode called schistosomes that affects humans and animals worldwide. Schistosomiasis endemically exists in developing countries. Inflammatory responses elicited in the early phase of infection represent the rate limiting step for parasite migration and pathogenesis and could be a valuable target for therapeutic interventions. Prostaglandin E2 (PGE2) and interleukin (IL)-10 were found to be differentially affected in case of immune-modulation studies and cytokine analysis of hosts infected with either normal or radiation-attenuated parasite (RA) which switches off the development of an effective immune response against the migrating parasite in the early phase of schistosomiasis. Normal parasites induce predominantly a T helper 2 (Th2)-type cytokine response (IL-4 and IL-5) which is essential for parasite survival; here, we discuss in detail the downstream effects and cascades of inflammatory signaling of PGE2 and IL10 induced by normal parasites and the effect of blocking PGE2 receptors. We suggest that by selectively constraining the production of PGE2 during vaccination or therapy of susceptible persons or infected patients of schistosomiasis, this would boost IL-12 and reduce IL-10 production leading to a polarization toward the anti-worm Thl cytokine synthesis (IL-2 and Interferon (IFN)-γ).

## Life cycle of ***Schistosoma mansoni***

**Fig. 1:**
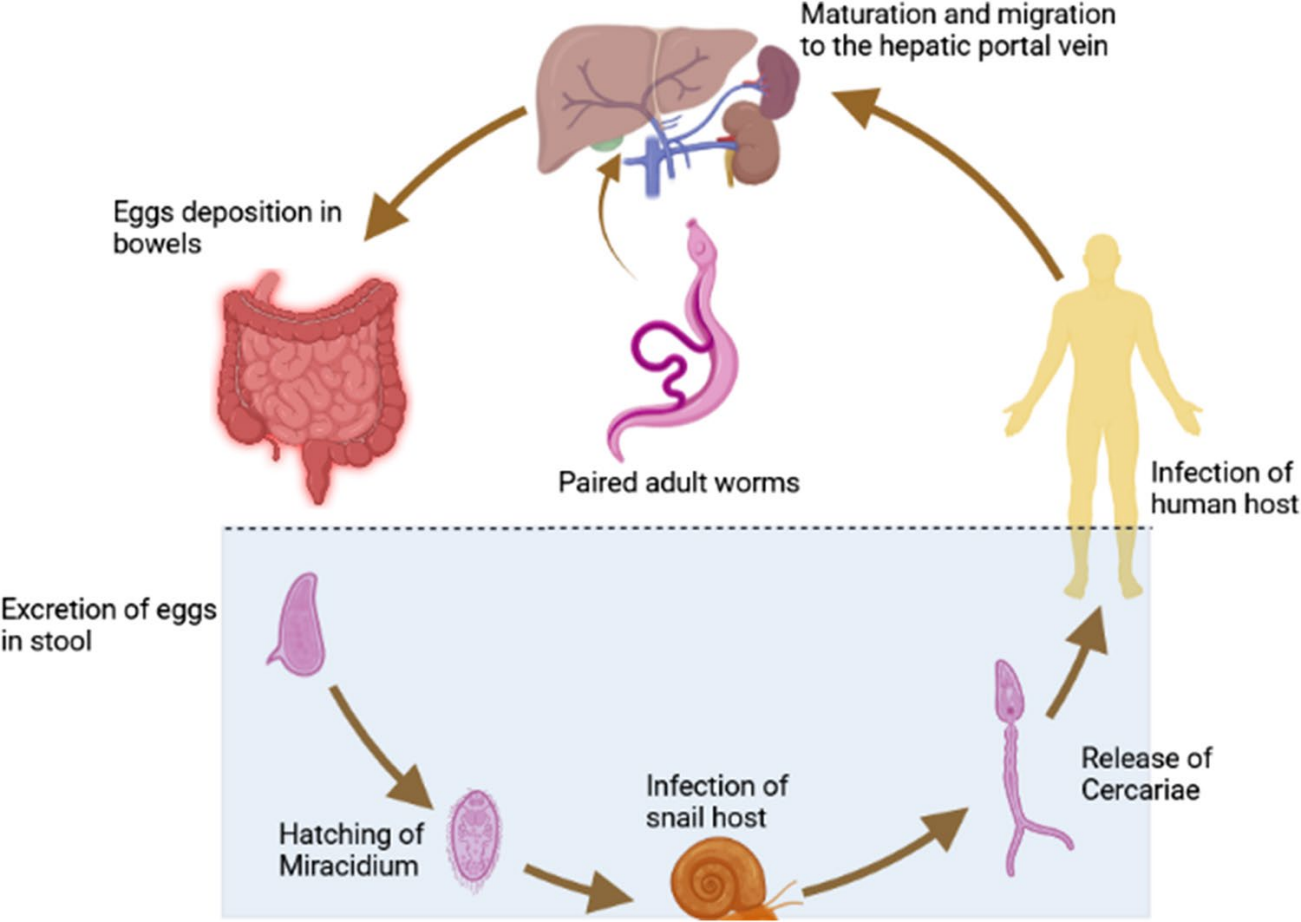
Life cycle of *Schistosoma mansoni*

Human schistosomiasis is acquired through the skin when the infective stage (cercariae) of the parasite penetrates the human skin exposed to contaminated water; the cercariae then convert into schistosomules and travel to the hepatic portal vein where they mature into adult worms. The male and female of adult schistosomes copulate in the mesenteric vein where the female worm lays around 250 ova per day, and then most of the ova get subsequently excreted with the feces. When the ova reach water, the larvae hatch, giving the miracidia that actively swim in water to find the snail intermediate host. The miracidia penetrate the snail’s soft tissue and transform into mature sporocysts that reproduce asexually to produce daughter sporocysts which turn into cercariae that are finally discharged into canals and rivers to infect humans and restart the cycle (McManus et al. 2018) (Fig. 1).

## Therapeutic modalities of schistosomiasis

Global community-based schistosomiasis control programs depending on massive drug treatment are crucial, particularly in endemic areas to minimize the debilitating manifestations of the parasite and decrease the morbidity. Safe and effective antischistosomal drugs are available and can be taken repeatedly especially in endemic areas to ensure complete elimination of infection when no more eggs are microscopically detected in urine or feces (Ross et al. 2002).

### Praziquantel

Praziquantel is the drug of choice for schistosomes, for more than 30 years, and praziquantel has been the main therapeutic choice for schistosomiasis. The anthelmintic effect of praziquantel is based on several hypotheses like inducing surface damage that causes the adult worms to detach from the venous walls, or antagonism of adenosine uptake (McManus et al. 2018). Praziquantel has no effect against the larval stages of the parasite or the immature schistosomes and cannot be used for prophylaxis (Ross et al. 2015).

### Artemisinin

Artemisinin which is a traditional antimalarial drug extracted from the medicinal plant *Artemisia annua* has shown profound activity against the schistosoma’s larval stages and immature schistosomula and can be used for chemoprophylaxis. Artemisinin and its derivatives are expected to exert toxic effect on schistosomes through interaction with hemin (formed from haem of human hemoglobin) inside the parasite (Saeed et al. 2016).

### Myrrh extracts

Myrrh is an oleo-gum resin, obtained from the stem of various *Commiphora spp.* (Burseraceae) and has been recently used against schistosomiasis. Pharmaceutical products of myrrh extracts showed potent antischistosomal activity with minimum side effects. The proposed mechanism of action of myrrh is the induction of tegumental damages and ulceration of the adult worms (El Ridi and Tallima 2013).

## Attenuated vaccine model

Larval stages of the parasite (cercariae) penetrate the skin and remain up to 72 h before traveling to the lungs. This stay in the skin gives the immune system enough chance to mount an efficient inflammatory reaction against the parasite. The host fails, however, to provoke any tissue response against schistosomulae residing in the skin (Skála et al. 2014). On the other hand, γ-irradiation-attenuated parasite infection stimulates a remarkable inflammatory response in the skin, leading to disrupted parasite migration (Winkel et al. 2019). This defect in the migration correlates with the promotion of IFN-*γ*-generating putative effector cells in the skin and draining of lymph nodes (García Nores et al. 2018). Interestingly, a challenge-related infection with normal parasites decreases the skin response to IFN-*γ* and the drainage of lymph nodes (Perrone Sibilia et al. 2019). This suggests that in immune individuals, a normal infection can impede a protective immune response against the parasite.

## PGE2 and parasite survival

**Fig. 2:**
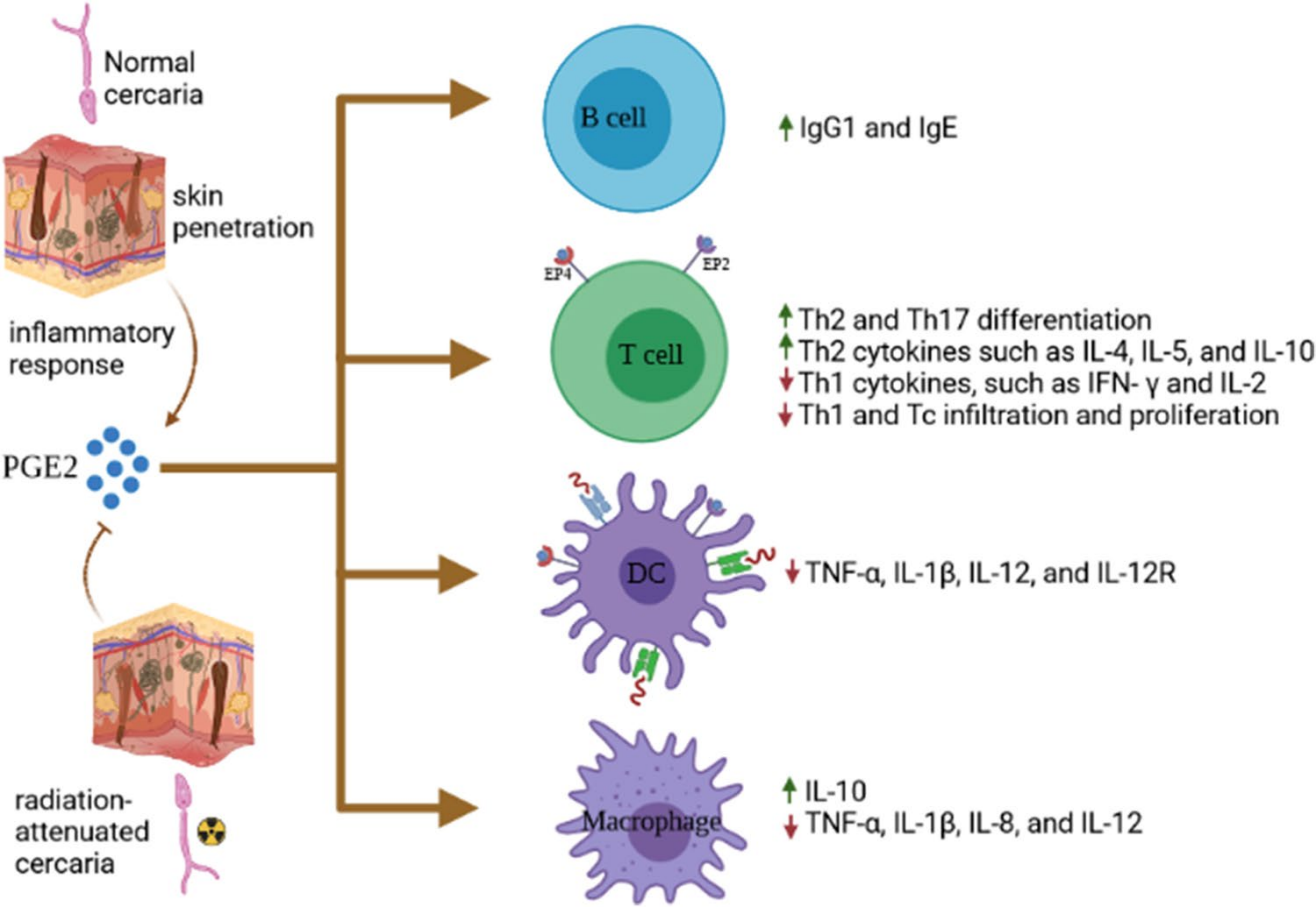
Influences of Prostaglandin E2 on immune cells and induced immune responses

Inflammation is an innate host response against injury or any pathogen (Abd El-Fattah et al. 2022; El-Rous et al. 2021). Prostaglandins are physiologically involved in several processes, including modulation of immune function and inflammatory response (Cruz et al. 2020). Prostaglandins act near their site of secretion and exert their actions by binding to one or more of their four E prostanoid receptor subtypes—EP1, EP2, EP3, and EP4 (Toyoda et al. 2019). PGE2 is a master regulator of immune responses (innate and acquired), particularly during immune priming, and has wide and diverse influences on different immune cells (Fig. 2) (Martínez-Colón and Moore 2018). Production or initiation of PGE2 release in the skin could be a deceptive adaptive mechanism established by the parasites for survival in the host. In addition, downregulation of dermal inflammation may be due to the processing of IL-10 (Winkel et al. 2018). Rather than the nature of the antigen per se, local condition during antigen priming may determine the resulting Th cell phenotype (Turner et al. 2017). The types of antigen presenting cells (APCs) involved in the presentation of antigens and local development of IL-4 and IL-12 cytokines by lymphocytes and accessory cells are critical factors (Schuijs et al. 2019).

Exposure to γ-irradiation seems to impair PGE2 production by the larva and its capability to stimulate PGE2 release in human keratinocytes (Ramaswamy et al. 2000; Winkel et al. 2018). The poor ability of the RA larvae to induce PGE2 and IL10 has been proposed to be responsible for differential capacity for infection compared to normal larvae (Ramaswamy et al. 2000; Winkel et al. 2018). IL-10 is released early after exposure to normal larvae (El-Aswad et al. 2020). Likewise, the level of dermal inflammation and the provoked protective immunity are governed by IL-10/IL-12 balance (Hogg et al. 2003). PGE2-driven IL-10 production may regulate the immune-modulation of inflammatory responses in the infected animals (Fig. 2). After contact with the skin-free fatty acids, parasite-derived PGE2 is released, activating IL-10 production (Fouzder et al. 2019).

Mice infected with normal parasites revealed a cytokine response predominantly of the Th2 class (Winkel et al. 2018). IL-10 and IL-4 mRNAs are specifically enhanced within 16 h of infection (Knuhr et al. 2018). In both human and mice, IL-10 is an inhibitory cytokine that has been engaged in the downregulation of multiple immune responses against *S. mansoni* (Osada et al. 2017). Thus, by modulating Th1-type responses, this early IL-10 response can perform a critical role in parasite manifestation.

IL-10 and IL-1 receptor antagonists (IL-1ra) are effective anti-inflammatory regulators (Arlati 2019). IL-1ra is developed by the skin keratinocytes in response to excretory/secretory products of *S. mansoni* cercariae (Osada et al. 2015). Skin cytokine released after *S. mansoni* infection showed a significant drop in IL-1a and IL-1b levels, while IL-1ra levels significantly increased, which might be attributed to the inhibitory effect of IL-10 on both IL-1a and IL-1b and its stimulatory effect on IL-1ra (Jamieson 2017).

## Modulation of cytokine profile during early schistosomiasis and effects on cell and humoral-mediated immunity

Cell-mediated immunity (CMI) is a helpful immune response distinguished with an increased population of particular T-cells (Okeke and Uzonna 2019). In the presence of antigens, this expansion promotes the development of localized cytokines. Herein, we review the modulation in the cytokine profile during early schistosomiasis and the corresponding effects on cell-mediated immunity.

### Effects on T-cells

T-cell activity is regulated by PGE2. CD4^+^ cells are heavily investigated for the feedback of PGE2 on the modulation of cytokine release, apoptosis, and proliferation (Giera et al. 2018). The negative effect of PGE2 on T-cell proliferation is well addressed. Interestingly, EP receptors perform a substantial role in inhibiting T-cell proliferation due to decreased secretion of IL-2 (Wang et al. 2018). During mucosal T-cell inflammation, EP4 receptors are upregulated with a concomitant reduction in T-cell production of IL-2; depending on the maturation and activation state of the cell, PGE2 differentially regulates activities of mature resting and activated T-cells by modulating apoptosis (Sreeramkumar et al. 2016). PGE2 performs multiple regulatory roles on T-cells, including its potential effect on cytokine production; improvement of Th2-type responses; promoting the production of Th2 cytokines such as IL-4, IL-5, and IL-10; and suppressing the levels of Th1 cytokines, such as IFN- *γ* and IL-2 (Kaisar et al. 2018) (Fig. 2).

In general, T-cell-mediated immunity is essential for acquired resistance against schistosomes in mice (Tang et al. 2019). Moreover, this protection is shown to be mediated by macrophage activation (Zheng et al. 2020). These studies, along with investigation of cytokine activity, suggest that treatments that induce macrophage activation of Th1 cytokines (IFN-γ) could be beneficial in preventing schistosomiasis (McManus and Loukas 2008). Thus, by activating a delayed-type hypersensitivity reaction, we aimed to stimulate cell-mediated parasite immune killing. By blocking PGE2 receptors (EP2 and EP4), this delayed reaction was achieved to decrease IL-10 and increase IL-12 early after infection. Naive Th cells could be activated by such inhibition to differentiate into Th1 cells with subsequent memory T-cell formation. These actions ascending from blocking PGE2 receptors mimic the schistosome vaccine-mediated events (Table 1).

**Table 1:** Stimulatory effects reported upon inhibiting the actions of PGE2 on its receptors EP2 and/or EP4

Reported action	Reference
Stimulate DCs maturation and increase their antigens presenting capacity	Gierlich et al. (2020)
Enhance MHC class II protein expression in DCs, and macrophages enhance their ability to act as APCs by inducing T-cell proliferation and T-cell clonal expansion	Couture et al. (2019)
Activate Th1 cytokines such as IFN-*γ* and IL-2	Nakanishi (2018)
Enhance CD8^+^ T-cell proliferation	Sreeramkumar et al. (2012)
Enhance the release of IFN-*γ* by CD8^+^ T-cells	Nakanishi (2018)
Proliferation and stimulation of B-cells	Abdel-Ghany et al. (2015)
IL-12 production by DCs	Abdel-Ghany et al. (2015)
Upregulation of IL-12 receptor on macrophages	Sheppe et al. (2018)
Production of TNF-*α* in • Monocytes • Macrophages	Abdel-Ghany et al. (2015)
Production of ILs by macrophages • IL-1*β* • IL-8 • IL-12	Sheppe et al. (2018)

### Effects on antigen-presenting cells

APCs perform essential roles in B- and T-cell-mediated immune responses; professional APCs such as dendritic cells (DCs) and macrophages are operated and modulated by PGE2. Immature DCs engage antigens in peripheral tissues, causing their activation and eventual migration to lymphoid organs. DCs mature in lymph nodes into APCs capable of presenting antigens to and priming T-cells (Fu and Jiang 2018; Gierlich et al. 2020; Harris et al. 2002). In these events, PGE2 was proposed to play a key role. Based on the place of encounter, PGE2 has contradictory effects on the activation of DCs (Diao et al. 2021). When the DCs transfer to lymphoid organs, PGE2 exerts a negative role through inhibiting the maturation of DCs and their antigen-presenting capabilities (Harizi 2013). PGE2 also controls cytokine output by DCs, thereby modulating subsequent immune responses. The differentiation of naive T-cells into Th2 cells was directly triggered by PGE2-primed DCs. High levels of IL-4 are provided by T-cells and no IFN-*γ* (Harris et al. 2002; Silva-Filho et al. 2014).

Lastly, PGE2 regulates the release of cytokines by activated macrophages. PGE2 downregulates IL-12 receptor expression and hinders macrophage development of TNF-α, IL-1β, IL-8, and IL-12 (Kalim and Groettrup 2013). Likewise, PGE2 dampens the secretion of TNF-*α* by LPS-treated peritoneal macrophages (Ikegami et al. 2001). Studies of zymosan-treated peritoneal mouse macrophages show that PGE2 induces TNF-*α* downregulation and IL-10 upregulation by means of EP2 and EP4 receptors (Chu et al. 2015). Thus, PGE2 upregulates type-2 responses in macrophages. PGE2 is generated by macrophages in large amounts, mostly in response to pro-inflammatory mediators such as IL-1 and LPS. Consequently, PGE2 may work as an autocrine feedback regulator since it positively regulates its own expression by upregulating COX-2 expression (Klein et al. 2007) (Fig. 2).

For DCs to operate as potent APCs during activation of immune responses, effective expression of MHC class II molecules is necessary. In DCs and macrophages, PGE2 constrains MHC class II expression and restricts their capability to function as APCs by inducing T-cell proliferation. Specific agonists were used for each EPR subtype to establish the subtype of EPRs implicated in PGE2-negative actions on MHC class II, and their findings indicated the participation of EP2 and EP4Rs in this inhibitory pathway regulated by PGE2 (Draijer et al. 2016; Harizi et al. 2003, 2001).

**Fig. 3:**
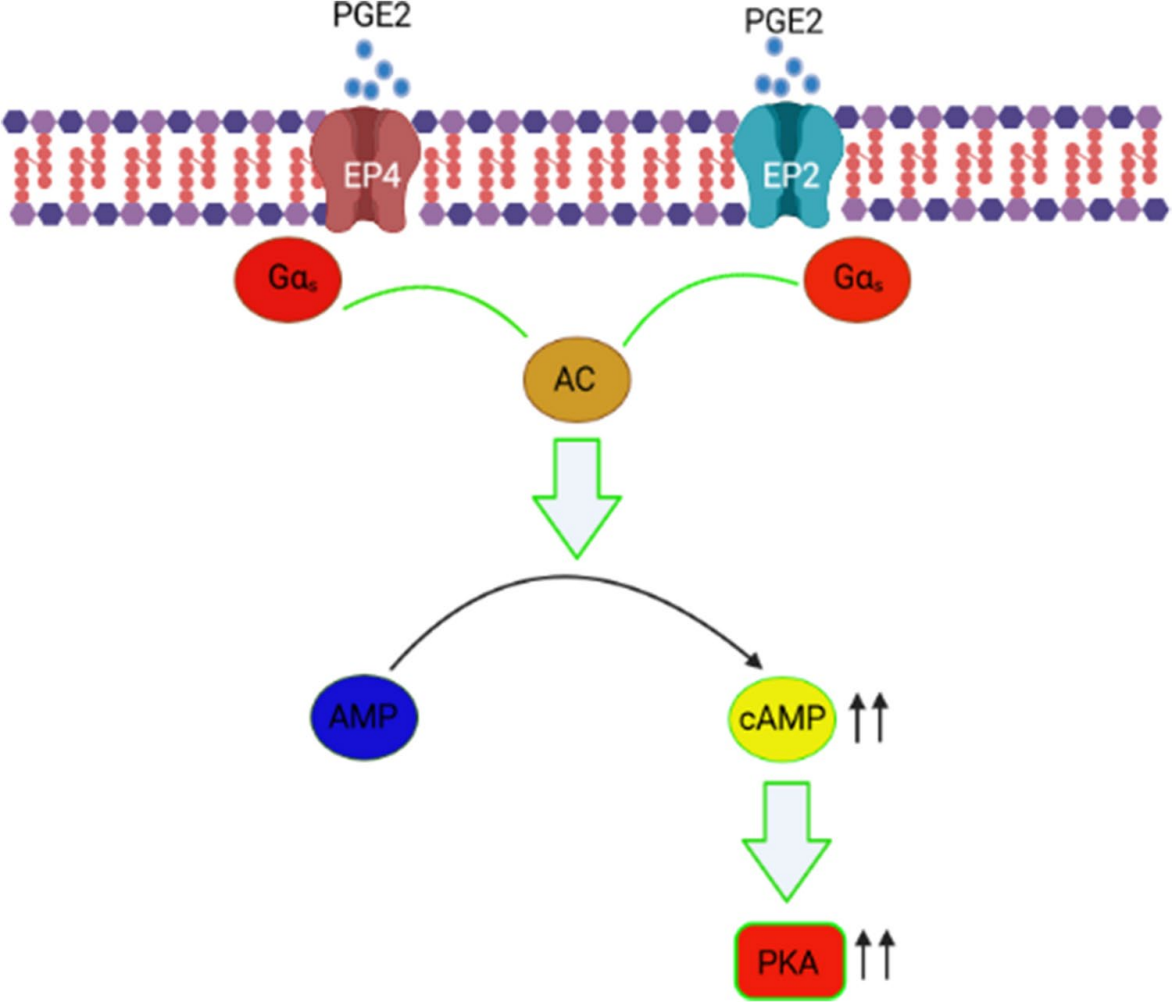
Prostaglandin E2/ E prostanoid receptors (EP2 and EP4) and downstream cAMP signaling

Eicosanoids, such as PGE2, differently regulate the capacity of DCs to produce cytokines (Esser-von Bieren 2017). PGE2 binds to DCs EP2R and/or EP4R that activate G-alpha protein, resulting in a surplus of cAMP required to release endogenous IL-10 (Fig. 3). IL-10 inhibits the release of other cytokines, such as IL-12. In addition, the expression of MHC class II molecules in DCs is inhibited, reducing their ability to function as competent APCs. In monocytes, the stimulation of endogenous IL-10 by cAMP provoking substances such as PGE2 has been detected as well (MacKenzie et al. 2013; Schulke 2018).

IL-12 is an initiation cytokine produced by APCs and is a principal mediator of CMI. Additionally, it might augment protection against infection (Mendez-Samperio 2010). IL-12 stimulates the release of IFN-*γ*, TNF-*α*, and GM-CSF, which leads to the transitions of macrophages, NK cells, and naive Th cells into Th1 cells (Ma et al. 2015).

### PGE2 immune-suppressing effect

Several findings suggest that PGE2 works as immunosuppressor by declining T-cell proliferation, IL-2 production, and IL-2 receptor expression (Salimu et al. 2017). Thus, PGE2 blunts T lymphocyte proliferation, an essential step in the clonal expansion of T-cells. It prevents IFN-alkaline release as well. PGE2 inhibits Th1 cytokine development, converting the immune response to the cytokine-type Th2 profile (Al-Taei et al. 2017).

### Effects on B-cells

In comparison to its effects on mature B-cells, PGE2 inhibits the growth and maturation of immature B-cells. PGE2 specifically induces immature B-cell apoptosis. PGE2-induced apoptosis is vulnerable to isolated neonatal mice B-cells, whereas mature mice isolated B-cells are not affected. In the same context, B-cell differentiation into IgE-secreting plasma cells is mediated by PGE2 (Harizi et al. 2003) (Fig. 2).

## PGE2/IL-12 balance and regulation of Th2 response

IL-12 is a key activator of T-cell differentiation into the Thl subtype. In contrast, IL-12 suppresses the development of Th2 (Tait Wojno et al. 2019). The capacity of IL-12 to control the proliferation of T-cells and NK cells and their production of IFN-*γ* probably underlies its Thl-inducing activity. PGE2 and other cAMP-inducing agents affect T helper response in opposition to IL-12 (Saber et al. 2020). Synthesis of Thl cytokines (IL-2 and IFN-*γ*) is further susceptible to inhibition by PGE2/cAMP than Th2 cytokine production (IL-4 and IL-5) (Tait Wojno et al. 2019). Thl-producing cytokines could negatively control the activity of Th2. Selective inhibition of Thl cytokines by PGE2 could therefore lead to a dominant Th2 response (Li et al. 2013). Since monocytes during antigen presentation can secrete both PGE2 and IL-12, the balance of them could regulate T-cell differentiation into either Thl or Th2 cells (Zheng et al. 2016).

PGE2 activates adenylate cyclase (AC) after binding to its receptor, resulting in high cAMP levels and activation of protein kinase A (PKA) (Fig. 3). Increases in intracellular cAMP appear to be responsible for the effects of PGE2, because other cAMP-inducing agents, such as DBcAMP, similarly inhibit IL-12 synthesis (Kalinski 2012). The PGE2/cAMP pathway could regulate Th2 responses by two mechanisms: (i) direct action by reducing the release of Thl cytokines such as IL-2 and IFN-*γ* or (ii) minimizing IL-12 production by monocytes. IL-12 downregulation may be the extremely substantial influence of PGE2 on the growth of T helpers and is more vulnerable to PGE2 inhibition. Thus, in deciding whether a Thl or Th2 response would prevail, a balance between IL-12 and PGE2 secretion by antigen-presenting monocytes is essential (Kalinski 2012).

## Non-steroidal anti-inflammatory drugs and possible indirect antischistosomal effects

Non-steroidal anti-inflammatory drugs (NSAIDs), e.g. diclofenac and ibuprofen, were previously investigated in experimental schistosomiasis caused by *S. mansoni*, where arachidonic acid metabolites participate in the pathogenesis (Nessim and Mahmoud 2007). Early treatment with diclofenac or ibuprofen led to a significant decrease in worm loads. No significant change was observed when treatment was delayed to 28 days post-infection.

Selective COX-2 inhibition by NS-398 alters EPR expression in mouse M-1 cells (Nasrallah et al. 2001). In agreement with other investigators, the addition of NS-398 to DCs specifically downregulates the expression of prostanoid receptors EP2R and EP4R, but not EP1R and EP3R. These outcomes indicate the existence of robust links between COX-2 and EP2R/EP4R expression and suggest that endogenous COX-2 produced PGE2 that could affect DCs by intensifying EP2 and EP4R expression (Ikegami et al. 2001; Nasrallah et al. 2001).

## Lung protective response

Learning from the schistosome vaccine model, immune priming in skin and sdLNs together with immune priming in the lung and lung draining lymph nodes is essential for acquired protection. This action ensures that immune response is largely dependent on Th1 cells (Cicchese et al. 2018).

The immune effector mechanism in the lungs is a cell-mediated DTH response that involves the formation of a tight focus of mononuclear cells and PMN leukocytes around embolized larvae (Chaplin 2010). CD4^+^ T-cells with Th1 criteria that secrete abundant INF-*γ* are a major component of the infiltrate. INF-*γ* has a substantial role in priming of macrophages/monocytes to release TNF-*α* (Cavalcanti et al. 2012). These cytokines act synergistically on host responses to infectious pathogens and are also involved in recruiting leukocyte to inflammation sites (Table 2). The immune response might involve an accumulation of leukocytes into a tight microenvironment that physically hinders the parasite migration in the vascular bed of lungs until the parasite expires (Costain et al. 2018).

**Table 2:** Collective stimulatory effects reported upon subsequent Th1-mediated response and their consequences on the parasites and helminths

Reported action	Reference
Macrophage-mediated pathogen killing	Muraille et al. (2014)
CD8 + lymphocytes maturation into cytotoxic T-cells	Uzhachenko and Shanker (2019)
Recruitment of macrophages by releasing TNF-α and chemokines (e.g. macrophage chemotactic factor-1) that operate on vascular endothelial cells to invite macrophages to the site of infection	Tang et al. (2020)
The production of IL-12 by macrophages, which drive further development of Th1 cells and IFN-*γ* secretion	Hamza et al. (2010), Lyakh et al. (2008)
Macrophage-mediated generation and secretion of toxic oxygen products and neutral proteases to kill helminths	Uzhachenko and Shanker (2019)
The generation and secretion of nitric oxide that has a role in killing pathogens	Schairer et al. (2012)
Generation and secretion of chemokines that act on vascular endothelial cells to attract other neutrophils to the site of infection. This action kills pathogens via secreting toxic metabolites, proteolytic enzymes for enzymatic digestion and other mechanisms	Uzhachenko and Shanker (2019)

In the schistosome vaccine model, heavily immune C57BL/6 mice with a Th1 bias had mostly mononuclear cell infiltrates that constituted condensed cores. On the contrary, poorly immune IFN-*γ*R^−/−^ mice with a Th2 bias comprised numerous eosinophils and lymphocytes in their pulmonary infiltrate, with large, diffuse foci that were loosely constituted and ineffective in trapping schistosomulae (Molehin et al. 2016). The extent of IFN-*γ* release determines the level to which CD4^+^ effector T-cells group into compact foci, maximizing their ability to trap migrating schistosomulae (Angeles et al. 2020; Hambrook and Hanington 2021). One obvious action of IFN-*γ* is the activation of macrophages for cytotoxic killing. Furthermore, the function of IFN-alpha in lung responses is to activate intercellular adhesion between leukocytes that are the target of the effector (Gomez et al. 2015). Indeed, alveolar macrophages are the first line of protection against pathogenic organisms in the lung and can induce cytokine overabundance, including chemokinesis (monocyte chemo-attractant protein-1 (MCP-1), macrophage inflammatory protein-1a (MIP-1*α*)), pro-inflammatory cytokines (IL-1, IL-6, and TNF-*α*) and immune-regulatory cytokines (IL-10, IL-12) (Angeles et al. 2020, Costain et al. 2018, Hambrook and Hanington 2021).

## Concluding remarks

Blocking PGE2 receptors (EP2 and EP4) in the early schistosomiasis would limit IL-4 and IL-10 production. IL-4 is the typical Th2 cytokine that skews naive T-cells into Th2 cells and constrains macrophage stimulation. IL-10 limits lymphocyte release of IFN-γ (Th1 cytokine) by T- and NK cells, minimizes T-cell proliferation, and is a powerful inhibitor of monocyte/macrophage functions and IL-12. Th1 cytokine response represents the protective immune response during schistosome infection. IL-12 production, the crucial cytokine for Th1 differentiation in general, is also upregulated by blocking these two receptors (EP2R and EP4R), which contributes to activation of inflammation, activation of naive T-cells, and their differentiation and polarization into Th1 cells. In conclusion, normal larvae do not initiate significant levels of immunologically mediated protection. Taken together, we propose that neutralizing the initial peak of PGE2 release in the skin by the parasite leads to diminished IL-10 and amplified IL-12 responses (Abdel-Ghany et al. 2015).

## Limitations and future perspectives

No vaccine is currently available to give a long-term protection against any of the human schistosomes. To identify a suitable vaccine candidate, a major condition is to select key schistosome molecules in the live parasite that could be recognized by the host immune system and are crucial for the parasite survival. Such vaccinable molecules may, for example, operate in parasite migration, immune avoidance, nutrient uptake or attachment. More than 100 schistosome vaccine antigens have been recognized; 25% of the recognized vaccines have displayed some level of protection in the murine model of schistosomiasis, and few of them are under clinical trials in humans (Tebeje et al. 2016).

Directing the immune response into Th1-biased response during early schistosomiasis may enhance the protective effects of the schistosome vaccines. In fact, a Th1-driving adjuvant such as IL-12 is effective in augmenting the efficacy of some defined vaccines up to 90% protection against schistosomiasis in murine models (Lebens et al. 2004; Santos et al. 2019; Stephenson et al. 2014; Tebeje et al. 2016).

Collectively, our review spots light on immunological interventions for augmenting the efficacy of some defined vaccines or treatments against schistosomiasis during migration phase.

## Data Availability

Not applicable. **Ethical approval and consent to participate.** Not applicable.
